# Detection of Emodin Derived Glutathione Adduct in Normal Rats Administered with Large Dosage of Polygoni Multiflori Radix

**DOI:** 10.3389/fphar.2017.00446

**Published:** 2017-07-06

**Authors:** Li-Long Jiang, Dong-Sheng Zhao, Ya-Xi Fan, Qiong Yu, Ping Li, Hui-Jun Li

**Affiliations:** State Key Laboratory of Natural Medicines, China Pharmaceutical UniversityNanjing, China

**Keywords:** Polygoni Multiflori Radix, liver injury, glutathione depletion, reactive metabolite, emodin-glutathione adduct

## Abstract

Polygoni Multiflori Radix (PMR) has been commonly used as a tonic in China for centuries. PMR-associated hepatotoxicity has been drawing increasingly more attention in recent years in parallel with its wide utilization. Anthraquinones (AQs) are recognized as the main hepatotoxic components in PMR. However, the exact underlying mechanism of AQs poisoning is still not fully understood. Herein, we proposed a hypothesis that metabolic activation of AQs such as emodin was involved in PMR-induced liver injury, AQs followed to generate the electrophilic reactive metabolites and subsequently formed covalent adduct with cellular nucleophiles in the liver to exert hepatotoxicity. In the present study, the link of cytotoxicity of PMR in primary human hepatocytes and the depletion of glutathione (GSH) was investigated by MTT assay and UHPLC-QqQ-MS/MS analysis. The results showed that PMR depleted GSH and therefore induced cytotoxicity. Then, emodin-GSH adduct was identified in bile of liver injured rats after intragastric administration of PMR or emodin with the aid of UHPLC-QTOF-MS/MS method. Our findings not only provided confirmative evidence that the mechanism of hepatotoxicity induced by AQs in PMR involved key metabolic steps, but also revealed that emodin-GSH adduct had potential to be further developed as a sensitive and traceable biomarker for the assessment of PMR-induced liver injury.

## Introduction

Polygoni Multiflori Radix (PMR), derived from the dried root of *Polygonum multiflorum* Thunb. (Family Polygonaceae), has been commonly used as a Chinese herbal medicine in clinical practice for health promotion and disease treatment for thousands of years, with the Chinese name “Heshouwu” (Lin et al., 2015a). It is traditionally valued for hair-blacking, liver and kidney-tonifying and anti-aging effects (Lin et al., 2015c). However, PMR-induced liver injury in clinic has been constantly reported in recent years (Yu et al., 2011). The earliest case of liver injury induced by PMR was recorded in 1996 in China (But et al., 1996). Since then, an increasing number of PMR-poisoning cases have been documented in many countries, including Australia, Italy, Holland, England, America, Korea, etc. (Park et al., 2001; Mazzanti et al., 2004; Panis et al., 2005; Cárdenas et al., 2006; Laird et al., 2008; Zhang et al., 2009; Jung et al., 2011). So far, a broad spectrum of chemicals such as stilbenes, anthraquinones (AQs), flavonoids, and phospholipids have been isolated from PMR (Lin et al., 2015b). Among these compounds, AQs including emodin, physcion, rhein, aloe-emodin, and chrysophanol, are commonly and conveniently believed to be primarily responsible for the PMR-associated hepatotoxicity, especially emodin, a predominant AQs occurring in PMR, is assumed to be one of the leading hepatotoxic component (Zhang et al., 2010; Li et al., 2012; Lin et al., 2015b; Lv et al., 2015; Ma et al., 2015). Nevertheless, the specific mechanism of AQs-induced liver injury is still unclear, which limits the establishment of effective approaches for diagnosis and treatment of liver injury induced by PMR.

It is well known that hepatotoxic effect of many xenobiotics requires metabolism (Tang et al., 1999; James et al., 2003; Peterson, 2013; Ruan et al., 2014). Hepatic metabolic activation of drugs to electrophilic reactive metabolites has been suggested to initiate the development of drug-induced liver toxicity (Guo, 2012). The reactive metabolites covalently bind to intracellular nucleophiles such as amino acids, glutathione (GSH), proteins, and nucleic acids, thereby triggering a series of pathologic alterations and causing liver injury (James et al., 2003; Fu et al., 2004; Lin et al., 2011). In general, conjugation with GSH is a major detoxification route for many xenobiotics (Chung et al., 1997; Chen et al., 2009), since this conjugation reaction protects cells against the harmful effects of reactive metabolites. On the other hand, the overproduction of electrophilic reactive metabolites will deplete GSH and eventually produce the toxicity (Kouzi et al., 1994; Shimizu et al., 2011). In this regard, the reactive metabolite-GSH conjugates have ever been developed as mechanism-based biomarkers to assess the toxicity of xenobiotics (Lin et al., 2011; Ruan et al., 2014).

Quinone compounds such as doxorubicin and menadione have been used as therapeutic drugs. However, clinical applications of these drugs are generally limited because of their hepatotoxicities. A prevailing mechanism of quinone-induced toxicity is that quinones as reactive electrophiles deplete GSH and covalently bind to proteins (Ishihara et al., 2011). AQs, structurally belonging to natural quinones, occur widely in many herbal drugs, including *Rheum palmatum* (Family Polygonaceae), *Cassia occidentalis* (Family Leguminosae), *Morinda citrifolia* (Family Rubiaceae), etc. Unfortunately, these AQ-containing drugs were documented to be toxic for human beings (Stadlbauer et al., 2008; Wang et al., 2009; Panigrahi et al., 2015). Despite many poisoning cases have been reported, there is still no clear consensus on the research of AQs-induced liver injury.

In the present study, for the purpose of verifying the depletion of GSH might be involved in PMR-induced liver injury, we tested the cytotoxic effect of PMR in relationship to its effect on the levels of intracellular GSH using MTT assay and ultra-high-performance liquid chromatography coupled to triple quadrupole mass spectrometry (UHPLC-QqQ-MS/MS) method. With repeated administration of 20 g/kg PMR for 3 weeks, significant elevations of serum total bilirubin (TBIL) and alkaline phosphatase (ALP) levels were observed, and massive necrosis was found in the liver with the histopathological analysis, indicating the liver injury induced by PMR. Furthermore, using ultra-high-performance liquid chromatography-quadrupole/time of flight mass spectrometry (UHPLC-QTOF-MS/MS) method, emodin-GSH conjugate was detected for the first time in the bile of liver injured rats after intragastric administration of PMR or emodin in corroboration of the mechanism proposed for AQs-induced liver injury initiated by metabolic activation.

## Materials and Methods

### Chemicals, Reagents and Animals

The decoction pieces of PMR were purchased from Bozhou traditional Chinese medicine market (Anhui Province, China). The samples were authenticated by Prof. Hui-Jun Li and deposited at State Key Laboratory of Natural Medicines (China Pharmaceutical University). Emodin was from Chengdu Must Bio-technology Co. (Chengdu, China). Dulbecco’s modified eagle medium (DMEM) was obtained from Gibco (Grand Island, NY, United States). Fetal bovine serum (FBS) was from Sijiqing (Hangzhou, China). GSH and GSSG assay kit and Bicinchoninic acid (BCA) assay kit were from Beyotime (Shanghai, China). Acetonitrile of HPLC grade was purchased from TEDIA Company (Fairfield, United States). Formic acid, acetic acid, and ammonium acetate (HPLC grade) were from ROE (Neward, New Castle, DE, United States). GSH and glutathione ethyl ester (internal standard, IS) were from Aladdin (Shanghai, China). Deionized water was prepared using a Milli-Q purification system produced by Millipore (Milford, MA, United States). All other reagents were analytical grade.

Male Sprague-Dawley (SD) rats, weighing 200-220 g, were provided by Sino-British Sippr/BK Lab Animal Ltd. (Shanghai, China). The animal studies were conducted in accordance with the Provision and General Recommendation of Chinese Experimental Animals Administration Legislation and the Instructive Notions with Respect to Caring for Laboratory Animals, and were approved by Animal Ethics Committee of China Pharmaceutical University and the Department of Science and Technology of Jiangsu Province [license number: SYXK (SU) 2016-0011]. The animals were housed under controlled conditions (temperature 22 ± 2°C, relative humidity 50 ± 10%) with a natural light-dark cycle for 1 week before the experiment was carried out.

### Preparation of Herbal Samples

Polygoni Multiflori Radix decoction pieces were cracked into small chunks, then the samples were extracted thrice under reflux each with 10 times of 75% ethanol for 3 h. The three extracts were combined and concentrated under reduced pressure, lyophilized, producing the dried PMR extract with a yield of 20.78%. The contents of the main constituents in PMR extracts were quantified by UHPLC method (Ma et al., 2012). The analytical results showed that the concentrations of emodin, physcion and 2,3,5,4′-tetrahydroxystilbene-2-*O*-β-D-glucopyranoside in PMR extract were 0.16, 0.13, and 5.69%, respectively. The lyophilized powders were re-dissolved and dispersed in 0.5% carboxymethyl cellulose sodium salt (CMC-Na) aqueous solution for intragastric administration to rats.

### Primary Human Hepatocytes Culture

Primary human hepatocytes were obtained from ScienCell (Carlsbad, CA, United States). The hepatocytes were recovered using the manufacturer’s protocol. Hepatocytes were grown in hepatocyte medium (ScienCell, Carlsbad, CA, United States) supplemented with 10% FBS, 1% hepatocyte growth supplement, 1% penicillin/streptomycin and maintained in a humidified atmosphere of 95% O_2_-5% CO_2_ at 37°C. The pH of the medium was maintained at 7.4. The hepatocytes were plated in 96-well round-bottom tissue culture plates to test cytotoxicity and plated in 6-well culture plates for GSH depletion assay. At the beginning of the experiment, when plated hepatocytes had reached about 85% confluence, the growth medium was removed from the wells, the hepatocytes were washed with PBS and fresh medium was used as base for treatment with the drugs.

### Cytotoxicity Assay in Primary Human Hepatocytes

MTT dye uptake in primary human hepatocytes in the presence and absence of PMR extract were determined. In brief, primary human hepatocytes (5 × 10^3^ cells/well) were seeded into 96-well plate and cultured for 24 h. The hepatocytes were then exposed to different concentrations of PMR extract. After 24 h of treatment, the media were removed and 100 μL MTT (0.5 mg/mL in medium) was added to each well. The plates were further incubated for another 4 h. Then, the media was removed from the wells and 100 μL of DMSO was added to each well. Finally the absorbance was taken at 490 nm after 10 min, on a micro plate reader. The percentage viability was calculated by comparing the absorbance of control and treated cells.

### GSH Depletion Assay in Primary Human Hepatocytes

Cellular GSH was determined using a UHPLC-QqQ-MS/MS method. Primary human hepatocytes were exposed for 24 h to either DMSO (control) or to increasing concentrations of PMR extract in 6-well culture plates. At the end of the incubation, the cell samples were washed twice with PBS before repeated freezing-thawing for three times in acetic acid-ammonium acetate buffering solution (pH = 3.5) and then centrifuged at 13000 rpm for 15 min at 4°C. Methanol (600 μL) was added to the supernatant (200 μL) to precipitate the protein, the supernatant was dried under nitrogen, reconstituted using 100 μL of ammonium acetate solution, vortex-mixed for 1 min, and centrifuged at 13000 rpm for 10 min at 4°C. The supernatant was injected into the UHPLC-QqQ-MS/MS system for analysis. The protein concentration was determined with BCA assay kit, and the GSH levels were expressed as ng/mL per mg protein.

### UHPLC-QqQ-MS/MS Analytical Condition and Method Validation

Samples were analyzed on an LC-MS/MS system consisting of an Agilent series 1290 UHPLC system and an Agilent 6460 triple quadrupole mass spectrometer (Agilent Technologies, Palo Alto, CA, United States). The separation was carried out on a Waters Xselect CSH C18 column (4.6 mm × 150 mm, 3.5 μm) at 30°C. Mobile phase adopted for this study was acetonitrile/water containing 0.1% (v/v) formic acid (10/90) and the flow rate was 0.6 mL/min. The mass spectrometer was operated in positive ion mode with electrospray ionization (ESI) source. The operating conditions were as follows: drying gas temperature, 325°C; drying gas flow, 10 L/min; sheath gas temperature, 300°C; sheath gas flow, 10 L/min; nebulizer pressure, 35 psi; capillary voltage, 3.5 kV. Quantitation was performed by multiple reaction monitoring (MRM) mode. Data collection and processing were conducted with MassHunter Workstation 05.00 (Agilent Technologies, United States).

Stock solution of GSH and IS were separately prepared in acetic acid-ammonium acetate buffering solution at the concentrations of 1 mg/mL. The GSH stock solution was diluted into a series of working solutions with the concentrations ranging from 5 to 20000 ng/mL, each containing 3.125 μg/mL of IS. Quality control (QC) samples with low, middle, and high concentrations were also prepared in the same way (10, 500, and 15000 ng/mL). All of the working solutions were stored at 4°C before use.

The UPLC-QqQ-MS/MS method was fully validated in terms of specificity, limit of quantification (LOQ), linearity, sensitivity, accuracy, precision, extraction recovery, matrix effect and stability, according to US FDA guidelines (United States Food and Drug Administration, 2001).

### Animal Protocol

Male Sprague-Dawley rats were randomly divided into two groups (control group and treatment group in different periods, *n* = 5) by intragastric administration with vehicle (1 mL/100 g rat) and PMR extract (20 g/kg) for 3 weeks. Blood, bile and liver samples were harvested at 1st, 2nd, and 3rd week, respectively. After the latest administration of PMR, urine from 0 to 24 h was collected and stored at –80°C until analysis. Blood samples were drawn at 24 h via the post-ocular vein, the plasma was generated by centrifugation at 4000 rpm for 15 min at 4°C and stored at -80°C before use. The rats were anesthetized with 20% urethane (0.5 mL/100 g, intraperitoneal injection), then the common bile ducts were cannulated with PE-10 tubing. The bile from 0 to 12 h was collected and stored at -80°C before use. At the end of the experiment, rats were sacrificed by cervical dislocation, and the livers in each group (*n* = 5) were harvested for histopathological examination. In a parallel study, emodin was suspended in 0.5% CMC-Na aqueous solution. The rats were treated with a single dosage of either vehicle or emodin (200 mg/kg) as control for the identification of GSH adduct. Urine from 0 to 24 h and bile from 0 to 12 h were collected and stored at –80°C until use. Blood samples were drawn at 24 h via the post-ocular vein for analysis.

### Biochemical Analysis and Histopathologic Examination

Blood samples collected at 24 h after the latest treatment of PMR were centrifuged at 4000 rpm for 15 min at 4°C. The alanine aminotransferase (ALT) activity, aspartate aminotransferase (AST) activity, total bile acid (TBA) concentration, TBIL concentration and ALP activity were determined by an automatic blood biochemical analyzer (Beckman Coulter LX20, United States). Liver function testing kits were supplied by Zhongda Hospital (Nanjing, China).

The rat liver tissues were fixed in 10% neutral buffered formaldehyde solution, paraffin-processed, and sectioned at 4 μm. The sections were stained with haematoxylin-eosin (HE) and examined for histopathological changes under the microscope (Olympus DX45, Japan).

### Detection of Emodin-GSH Conjugate in Plasma, Urine and Bile

Methanol (threefolds) was added to plasma, urine and bile to precipitate the protein, then vortex-mixed for 1 min and centrifuged at 13000 rpm for 10 min. The supernatants of plasma and urine samples were dried under nitrogen and dissolved in 100 μL of methanol for UPLC-QTOF-MS/MS analysis. The supernatant (2 μL) of bile samples was directly injected into the UPLC-QTOF-MS/MS system for analysis.

Biological specimens were analyzed on an Agilent 6530 QTOF-MS/MS with ESI in negative-ion mode. The chromatographic separations were achieved on a Shimadzu VP-ODS column (250 mm × 4.6 mm, 5 μm). The mobile phase of water containing 0.1% (v/v) formic acid (solution A) and acetonitrile (solution B) was used with a gradient elution as follows: 0–55 min, 8–45% B; 55–57 min, 45–100% B; 57–65 min, 100% B. The UHPLC flow rate was 0.8 mL/min. The operating conditions for ESI mass spectrometry were as follows: drying gas temperature, 320°C; capillary voltage, 3500 V; drying gas flow rate, 10 L/min; nebulizing gas pressure, 35 psi; sheath gas temperature, 350°C; sheath gas flow, 11 L/min; fragmentor voltage, 125 V. The collision energies were 30 and 60 V. The molecular mass was accurately measured in negative-ion mode to analyze emodin-GSH conjugate. The characteristics of ion pairs for emodin-GSH conjugate were *m*/*z* 574 → 272.

### Statistical Analysis

The statistical analysis was performed using a one-way ANOVA with the Dunnett’s *post hoc* test to determine the significance of the differences between the individual groups. A value of *p* < 0.05 was considered as statistically significant.

## Results and Discussion

### Effect of PMR on Hepatocytes Viability and Cellular GSH

**FIGURE 1:**
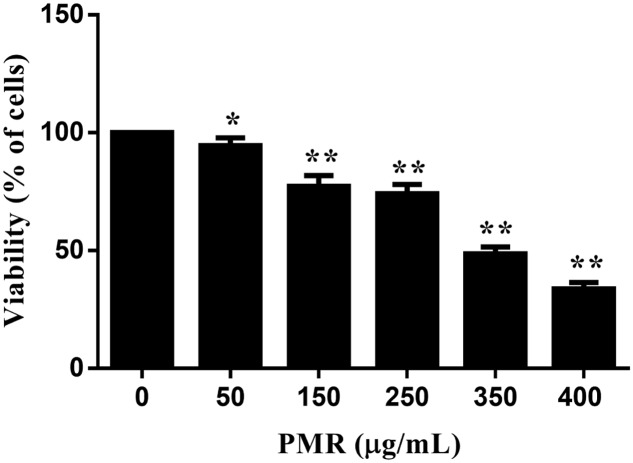
Cell viabilities of primary human hepatocytes exposed to Polygoni Multiflori Radix (PMR) for 24 h. PMR inhibited the cell viabilities in a dose-dependent manner. Data are presented as means ± SD of three independent experiments. ^∗^*p* < 0.5, ^∗∗^*p* < 0.01 vs. control.

MTT assay was used to estimate the cytotoxicity of PMR to primary human hepatocytes. As shown in **Figure 1**, cell viabilities were decreased in a dose-dependent manner after exposure to different doses of PMR (50, 150, 250, 350, and 400 μg/mL) for 24 h. The IC_50_ values of PMR were 331.30 μg/mL. These results revealed the *in vitro* hepatotoxicity of PMR.

As the cytotoxicity was observed with the treatment of PMR, we further investigated the relationship between GSH depletion and the cytotoxicity in primary human hepatocytes, with the aid of UHPLC-QqQ-MS/MS method. In order to achieve a good resolution, the chromatographic conditions were optimized. Finally, the MRM transitions were monitored at *m*/*z* 308.10 → 76.10 and *m*/*z* 336.10 → 76.10 for GSH and IS, respectively. The fragmentor voltage values set for GSH and IS were 110 and 100 V, and the collision energies were 25 and 30 V, respectively.

**FIGURE 2:**
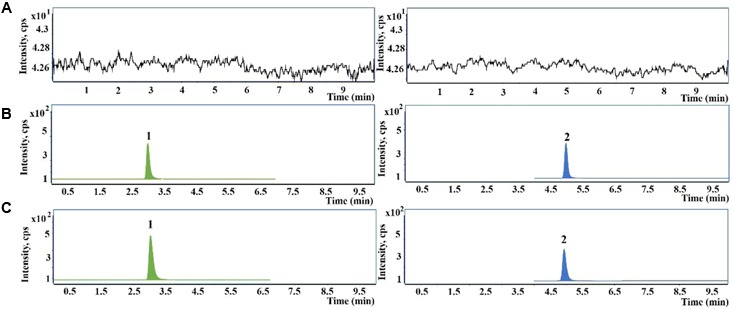
Representative chromatograms of glutathione (GSH) and glutathione ethyl ester [internal standard (IS)] in primary human hepatocytes samples. **(A)** Blank hepatocytes sample; **(B)** blank hepatocytes sample spiked with GSH and IS; **(C)** hepatocytes sample treated with PMR. 1, GSH; 2, IS.

The representative UHPLC-QqQ-MS/MS MRM chromatograms of GSH and IS are shown in **Figure 2**. The calibration exhibited satisfactory linearity, ranging from 5 to 20000 ng/mL. The LOQ of GSH was 1.25 ng/mL. The intra- and inter-day precision and accuracy were almost less than 15%, indicating the accuracy and precision of the method were acceptable for the quantitative analysis. The extraction recoveries of GSH were 91.50–94.73%, respectively. The matrix effect was also found to be satisfactory.

**FIGURE 3:**
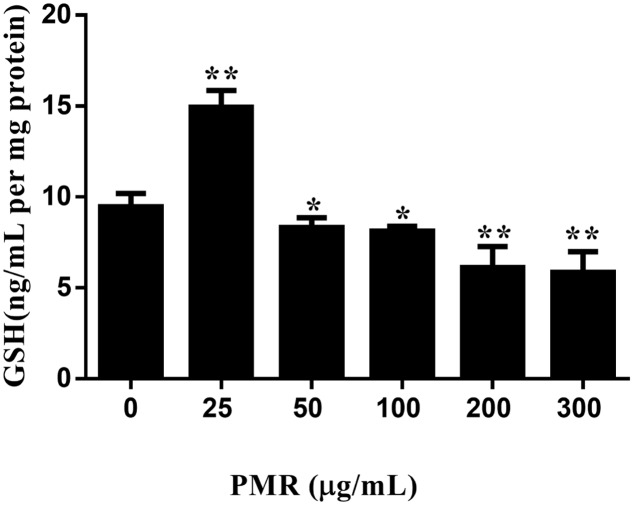
Depletion of GSH induced by PMR in primary human hepatocytes *in vitro*. PMR depleted cellular GSH in a dose-dependent manner when PMR concentration was greater than 50 μg/mL. Data are presented as means ± SD of three independent experiments. ^∗^*p* < 0.05, ^∗∗^*p* < 0.01 vs. control.

Hence, the intracellular GSH levels were quantified by the newly developed UHPLC-QqQ-MS/MS method and the quantitative results are illustrated in **Figure 3**. As shown in **Figure 3**, treatment with the concentration of 25 μg/mL PMR resulted in an increase in GSH level compared to control group, indicating a protective effect of PMR in low concentration, whereas dramatic GSH depletion was found to occur in the concentrations of 50, 100, 200, and 300 μg/mL. At the concentration of 50 μg/mL, PMR significantly decreased GSH level by 12.05% as compared to control group. All of these results suggested that GSH depletion might be a critical event in the mechanism of PMR-induced liver injury.

### Evaluation of Hepatotoxicity of PMR

**FIGURE 4:**
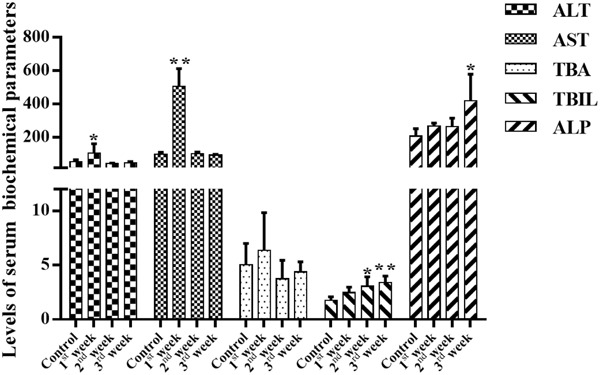
Effects of PMR on the levels of serum biochemical parameters. Total bilirubin (TBIL) concentrations were increased in a time-dependent manner, and a significant increase in plasma alkaline phosphatase (ALP) levels was observed after 3 weeks treatment of PMR, which might be more sensitive than alanine aminotransferase (ALT), aspartate aminotransferase (AST), and total bile acid (TBA). Data are expressed as mean ± SD (*n* = 5). ^∗^*p* < 0.05, ^∗∗^*p* < 0.01 compared with control groups.

**FIGURE 5:**
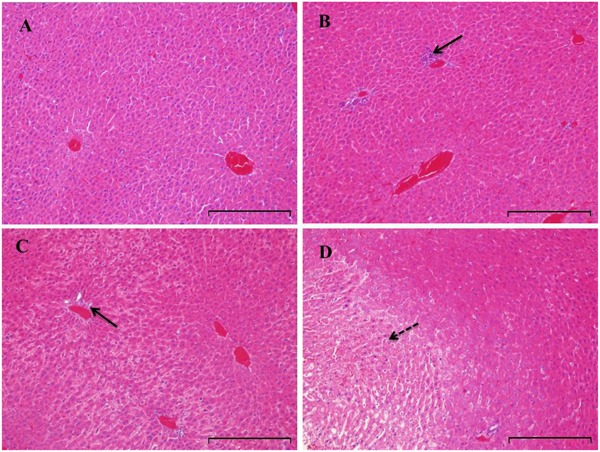
Histopathologic evaluation (H&E staining) of liver tissues obtained from rats treated with PMR (*n* = 5). Original magnification was 200× for each sample. Rats were treated with vehicle **(A)**, PMR for 1 week **(B)**, PMR for 2 weeks **(C)**, PMR for 3 weeks **(D)**. Bar = 200 μm. After 3 weeks treatment of PMR, necroses were observed in the livers of rats, indicating the liver injury induced by PMR. Inflammatory cell infiltration and necrotic region were indicated by solid and dotted arrows, respectively.

**FIGURE 6:**
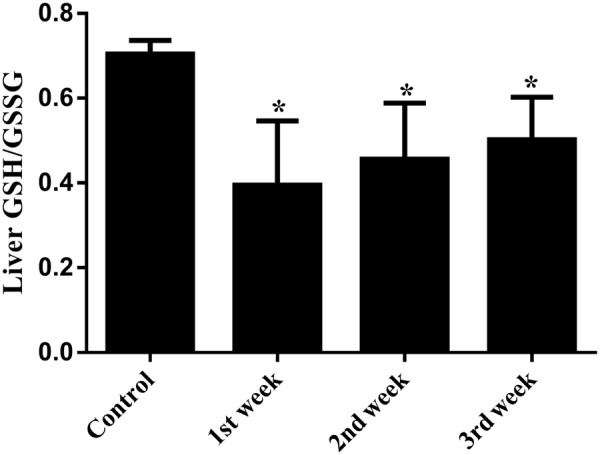
Hepatic GSH/GSSG ratio of PMR-treated rats. During the treatment of PMR, GSH/GSSG ratios were significantly decreased in livers of rats. ^∗^*p* < 0.05 compared with the control group.

Serum liver enzymes are the most commonly used biomarkers for diagnosis and assessment of hepatic diseases in clinic (Abboud and Kaplowitz, 2007). In the present study, five conventional biomarkers of liver injury, namely ALT activity, AST activity, TBA concentration, TBIL concentration and ALP activity, were examined to evaluate the liver injury induced by PMR. As can be seen in **Figure 4**, the levels of TBA did not show significant changes between the control group and the treatment groups. Intragastric gavage of PMR caused significant elevations of serum ALT and AST activities at the 1st week (serum ALT and AST: 103.00 and 504.67 U/L for treatment group; 52.40 and 96.25 U/L for control group). No obvious alterations of serum ALT and AST activities were observed at the 2nd and 3rd weeks (serum ALT and AST: 40.60 and 100.00 U/L at 2nd week; 45.25 and 92.25 U/L at 3rd week). The TBIL concentrations showed a time-dependent increase during the treatment of PMR, with significant alterations at 2nd and 3rd weeks compared to the control group (3.05 and 3.40 μmol/L at 2nd and 3rd weeks; 1.77 μmol/L for control group). Meanwhile, the administration of PMR for 3 weeks showed a significant elevation of ALP levels when compared to the control group (418.75 U/L at 3rd week; 205.00 U/L for control group). The histopathologic evaluations (**Figure 5**) demonstrated that a small amount of inflammatory cells were observed in livers of PMR-treated rats at 1st and 2nd weeks (**Figures 5B,C**), while liver cell necrosis could be found after 3 weeks of PMR treatment (**Figure 5D**), demonstrating the insensitivity of conventional biochemical parameters in plasma like ALT, AST, and TBA for the indicating function of PMR-induced liver injury. Remarkably, TBIL and ALP might be more sensitive biomarkers during the process of liver injury induced by PMR. These findings were consistent with previous report (Tu et al., 2015; Zhao et al., 2017). Additionally, the patients often take multi-herb preparation containing PMR (Lei et al., 2015), leading to the uncertainty whether the liver injury is truly induced by PMR. It is clinically necessary to identify the traceable biomarkers for the diagnosis of PMR-induced liver injury. Meanwhile, the GSH/GSSG ratio in PMR-administrated liver was analyzed according to the technical manual of the detection kit. As shown in **Figure 6**, the GSH/GSSG were significantly decreased during 3 weeks, indicating that PMR administration depleted GSH in livers of rats, and slight increases were observed at the 2nd and 3rd weeks when compared to the 1st week, this would be considered as a compensatory response of rats. The result revealed that GSH depletion might be involved in PMR-induced liver injury.

### Identification of Emodin-GSH Adduct in Liver Injured Rats

**FIGURE 7:**
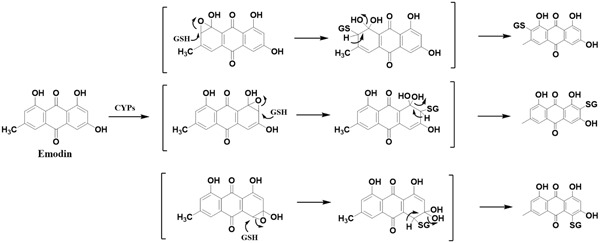
Proposed metabolic biotransformation of emodin and the generation of emodin-GSH adduct. CYPs mediated emodin bioactivation involved the formation of an electrophilic epoxide intermediate. Theoretically, the epoxidation reaction of anthracene ring occurred in multiple positions, resulting in the diversity of GSH conjugation of emodin.

Considering that quinones are easily subjected to metabolic activation (Wu et al., 2014), we speculate that AQs in PMR preferentially undergo phase I metabolism mediated by cytochromes P450s (CYPs) and form electrophilic epoxide intermediate metabolite, then covalently bind to nucleophilic GSH (He et al., 2015). Owing to the relative high abundance in PMR extract, emodin was exemplarily selected as model compound. **Figure 7** is the proposed bioactivation route of emodin-GSH.

**FIGURE 8:**
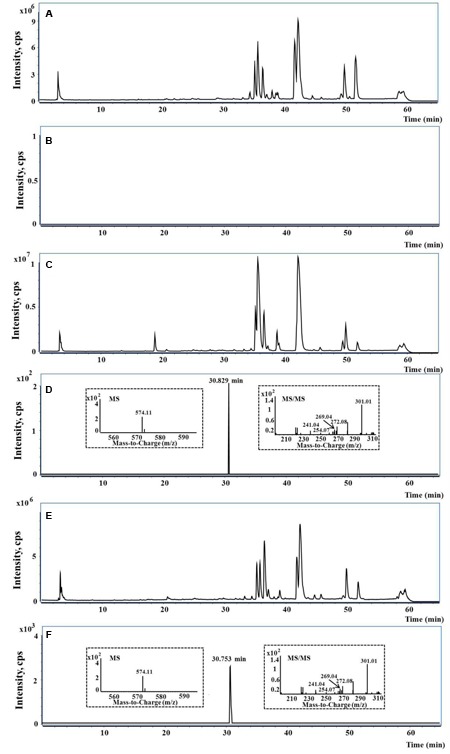
The representative UHPLC-QTOF-MS/MS chromatograms. **(A,B)** The typical total ion current (TIC), extract ion (EI) at *m*/*z* 574 in rat blank bile; **(C,D)** the typical TIC, EI at *m*/*z* 574 and MS/MS spectra in bile of rat treated with PMR; **(E,F)** the typical TIC, EI at *m*/*z* 574 and MS/MS spectra in bile of rat treated with emodin.

A UHPLC-QTOF-MS/MS-based analytical approach was employed to identify emodin-GSH adduct in bio-samples (**Figure 8**). Because more abundant mass information were acquired in negative mode than in positive mode, therefore we chose negative ion mode for structural characterization and identification. Consequently, the target ion ([M-H]^-^) was predefined at *m*/*z* 574. One extensive peak with *m*/*z* 574.11 at retention time of 30.8 min was observed in the extract ion spectra of bile samples. No such peak was detected either in the control group or in the plasma and urine of rats after intragastric gavage of PMR or emodin (data not shown).

**FIGURE 9:**
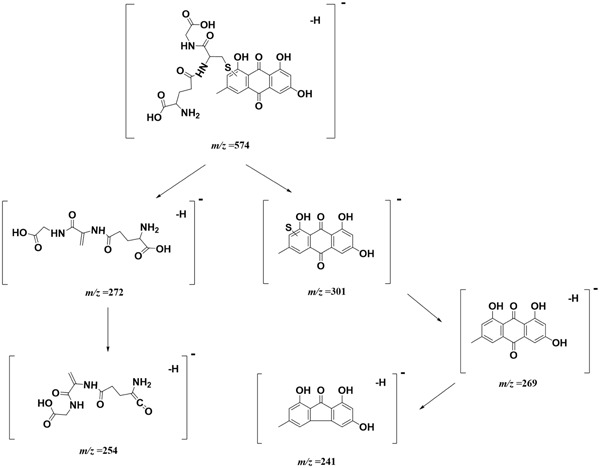
The proposed fracture way of emodin-GSH adduct.

Taking the advantage of QTOF-MS/MS, the accurate mass values of this peak were within the range of *m*/*z* 574.1109–574.1172, the errors of accurate mass of the tested samples were from 0.18 to 6.08 ppm compared with the exact mass of the target adduct (calculated mass 574.1137 for C_25_H_24_O_11_N_3_S). Furthermore, the characteristic fragmentation ions at *m*/*z* 301.01, 272.08, 269.04, 254.07, and 241.04 were observed from the MS/MS spectra of the precursor ion ([M-H]^-^) (**Figures 8D,F**). The product ions at *m*/*z* 301.01 and 272.08 were two complementary ions, corresponding to the moieties of sulfur-substituted emodin and γ-glutamyl-dehydroalanyl-glycine (Dieckhaus et al., 2005), respectively. The ions at *m*/*z* 269.04 and 241.04 were generated by successive neutral losses of sulfur atom (32 Da) and carbon monoxide (28 Da) from the ion at *m*/*z* 301.01, the ion at *m*/*z* 254.07 was ascribed to the loss of water (18 Da) from at *m*/*z* 272.08. Thus, the detected peak with *m*/*z* 574.11 was supposed as the emodin-GSH adduct. The detailed fragmentation pathway is shown in **Figure 9**.

### Significance of Emodin-GSH Adduct Detected in Bile of PMR-Induced Liver Injured Rats

Drugs are generally metabolized by a variety of chemical processes involving oxidation, reduction, and hydrolysis (phase I reactions) or glucuronidation, sulfation, acetylation, and methylation (phase II reactions) (Wilkinson, 2005). AQs underwent both phase I and phase II metabolism. Several phase I metabolites had been identified (Song et al., 2009), the phase II conjugates, especially glucuronides were the predominant existing forms of AQs *in vivo* (Wu et al., 2014). The phase II conjugation reaction was recognized as a detoxification step through increasing water solubility of AQs and enhancing their removal. As we know, GSH is a major intracellular antioxidant which is critical for preserving normal cellular redox balance and protecting the liver against oxidative stress (Chen et al., 2009). However, nucleophilic addition to GSH plays a key role in the detoxification reaction due to the nucleophilicity of GSH. In the present work, the detection of emodin-GSH adduct in bile of PMR-induced liver injured rats suggested that a portion of emodin underwent a crucial metabolic pathway known as epoxidation via hepatic drug-metabolizing enzymes, such as CYPs toward formation of epoxide intermediate (Peterson, 2013; He et al., 2015). The resulting epoxide subsequently attacked nucleophiles such as GSH, proteins, DNA and membranes that eventually induced liver injury.

Very recently, the hydroquinone intermediates of emodin were characterized in liver microsomes and the electrophilicity of emodin was determined (Qin et al., 2016), which supported the mechanism proposed in the present research that AQs-induced liver injury initiated by metabolic activation. And certainly, the specific metabolism of emodin *in vivo* especially phase I metabolic pathway associated with the toxic effects warranted further confirmation.

According to the histopathologic evaluations, inflammatory cell infiltration were observed in livers of PMR-treated rats at 1st and 2nd weeks, liver cell necrosis was observed after 3 weeks administration of PMR (**Figure 5D**), the emodin-derived GSH adduct could be detected from the 1st week during the administration of PMR, strongly suggesting that it had potential as an early sensitive and traceable biomarker exposure to PMR. Further study is to synthesize sufficient amounts of the chemical entity of emodin-GSH adduct that enables a dynamic quantification analysis to reflect the status and severity of injury. Notably, the emodin-GSH adduct was not detected in the plasma and urine, a similar observation was made in another research (Yang et al., 2014), this could be explained by the fact that the conjugated emodin was mainly excreted via bile (Bachmann and Schlatter, 1981) or the undetectable concentration of emodin-GSH adduct in blood or urine. On the basis of the detection of emodin-GSH adduct, the derivatization and detection of emodin-GSH conjugates or emodin-protein adducts in blood or urine deserve further investigation for clinical detection.

## Conclusion

To date, there is no clear consensus concerning to the hepatotoxic ingredients of PMR as well as the underlying mechanism. Some studies suggested that the hepatotoxicity of PMR was probably idiosyncratic in patients (Jung et al., 2011; Dong et al., 2013). A recent research demonstrated that combined treatment with lipopolysaccharide and PMR resulted in acute idiosyncratic liver injury in rats, and revealed that 2,3,5,4′-tetrahydroxy *cis*-stilbene-2-*O*-β-glucoside was closely associated with the idiosyncratic hepatotoxicity of PMR (Li et al., 2017). On the other hand, PMR would cause chronic hepatitis in general (Ma, 2013). The liver injury occurred from several weeks to several months after PMR administration. Our previous toxicokinetic studies about an excessive consumption of PMR disclosed that the 2,3,5,4′-tetrahydroxy *trans*-stilbene-2-*O*-β-glucoside could inhibit the glucuronidation of emodin in the phase II metabolism and bring about a chronically accumulative emodin exposure, ultimately reaching a threshold and resulting in hepatotoxicity (Ma et al., 2013, 2015). In this continued study of PMR-associated hepatotoxicity, we herein put forward a hypothesis that AQs followed metabolic activation to generate the electrophilic reactive metabolites and subsequently formed covalent adduct with cellular nucleophiles in the liver to exert hepatotoxicity. First of all, the relationship between hepatotoxicity and GSH depletion was established, which demonstrated the important role of GSH in regulating the PMR hepatotoxicity. Then, emodin-derived GSH adduct was identified in bile of PMR-treated rats, suggesting that the metabolic activation of AQs toward epoxide formation might be one of the prime triggers for liver injury. The detection of emodin-GSH conjugation also indicated that the GSH conjugate showed a potential to be developed as a sensitive and traceable biomarker for the diagnosis of PMR-induced liver injury. Taken together, this study will not only shed novel insight into revealing the underlying mechanism of PMR-induced liver injury, but also offer a significant clue for hepatotoxic research of other AQs-containing herbs.

## Author Contributions

H-JL and PL conceived and designed the experiments; L-LJ and QY performed the experiments; L-LJ and D-SZ analyzed the data; Y-XF and QY contributed reagents/materials/analysis tools; H-JL and L-LJ wrote the paper.
